# Immune Checkpoints as the Immune System Regulators and Potential Biomarkers in HIV-1 Infection

**DOI:** 10.3390/ijms19072000

**Published:** 2018-07-09

**Authors:** Maike Sperk, Robert van Domselaar, Ujjwal Neogi

**Affiliations:** 1 Division of Clinical Microbiology, Department of Laboratory Medicine, Karolinska Institutet, 141 86 Stockholm, Sweden; ujjwal.neogi@ki.se; 2 Department of Medicine Huddinge, Unit of Infectious Diseases, Karolinska Institutet, Karolinska University Hospital, 141 86 Stockholm, Sweden; robert.van.domselaar.1@ki.se

**Keywords:** biomarker, human immunodeficiency virus (HIV), immune checkpoint, programmed cell death protein 1 (PD-1), T cell exhaustion

## Abstract

Immune checkpoints are several co-stimulatory and inhibitory pathways that regulate T cell immune responses. Most of the discoveries about immune checkpoints were made in cancer research where inhibitory immune checkpoints cause immune exhaustion and down-regulate anti-tumor responses. In addition to cancer, immune checkpoints are exploited in chronic infectious diseases. In human immunodeficiency virus (HIV) infection, the immune checkpoint molecule called programmed cell death protein 1 (PD-1) has been determined as being a major regulatory factor for T cell exhaustion. Recent studies with antibodies blocking either PD-1 ligand 1 (PD-L1) or PD-1 show not only promising results in the enhancement of HIV-specific immune responses but even in reducing the latent HIV reservoir. Apart from the therapeutic target for a functional cure of HIV-1, immune checkpoint molecules might be used as biomarkers for monitoring disease progression and therapeutic response. In this review, we will summarize and discuss the inhibitory immune checkpoint molecules PD-1, cytotoxic T-lymphocyte-associated protein 4 (CTLA4), lymphocyte-activation gene 3 (LAG3), and T cell immunoglobulin and mucin-domain-containing-3 (TIM3) as well as the co-stimulatory molecules CD40L and CD70, including their role in immunity, with a particular focus on HIV infection, and being potential targets for a functional HIV cure.

## 1. Introduction

Immune checkpoints are the regulators of T cell immune responses in response to invading pathogens or mutated/overexpressed self-antigens by regulating the balance between co-stimulatory and inhibitory signals [1,2]. In the early 1990s, the first so-called immune checkpoint molecule, programmed cell death protein 1 (PD-1), was discovered and associated with apoptosis [3]. Since then this field of research has resulted in today’s application of immune checkpoint therapies. The “two-signal hypothesis” suggests that T cell activation requires two signals: first, a complex consisting of an antigen (Ag) and major histocompatibility complex class II (MHC II) has to be recognized by the T cell antigen receptor; and second, additional co-stimulatory signals are needed for full activation of naïve T cells [4]. The engagement of T cell surface receptor CD28 with B7 molecules (CD80 and CD86) on antigen-presenting cells (APC) was the first described co-stimulatory interaction [5]. Many more receptors and ligands with a broad diversity in expression, structure, and function have now been identified [6], rendering the two-signal hypothesis more complex. This hypothesis has become more extended as co-inhibitory interactions have been characterized. The spatiotemporal organization of both co-stimulatory and co-inhibitory signals is crucial for adequate T cell activation, proliferation, differentiation, and function. The physiological expression of immune checkpoints is essential for maintaining peripheral tolerance as well as impeding autoimmunity. Furthermore, expression is induced after T cell activation providing a negative feedback loop to suppress activated T cell responses. In that way, they modulate the duration and amplitude of a physiological immune response [1,2,6]. From a pathological point of view, inhibitory co-regulatory pathways can be up-regulated after chronic exposure to antigens, particularly in the tumor microenvironments (TME) in a variety of cancers and during chronic infections, inducing a state of antigen-specific T cell exhaustion. However, immune checkpoints differ in their mechanisms of actions and at which immune activation level they are acting. For example, cytotoxic T lymphocyte antigen 4 (CTLA4) counteracts the activity of co-stimulatory receptor CD28 and thus influences the early stage of T cell activation. On the other hand, PD-1 limits the effector function of activated T cells in peripheral tissues. As both co-stimulatory and co-inhibitory pathways consist of membrane-bound and soluble receptor-ligand pairs, they are promising drug targets in immune checkpoint therapy [1,7].

Although initially studied in the context of oncology, evidence is emerging that immune checkpoints also play crucial roles in viral infections, including human immunodeficiency virus (HIV) infections. Therefore, immune checkpoint molecules gain importance in HIV-1 research. Immune checkpoint therapies could appear as promising vital components of the complex therapeutic strategies aiming for a functional cure for HIV infection [8,9]. A functional cure means that HIV-infected patients, although still having the virus present in the body, constantly have a low-level viral load in the absence of antiretroviral therapy (ART). Studies have hypothesized that monoclonal antibodies that are blocking immune checkpoints may serve as latency-reversing agents (LRA) and may enhance the CD8+ T cell effector functions [10,11,12,13,14]. Changes in expression patterns and protein levels of PD-1 and other immune checkpoints might also serve as prognostic biomarkers in tuberculosis, malaria, and chronic viral infections.

In this review, we will summarize and discuss selected immune checkpoints as prognostic biomarkers in HIV-1 infection, their role in immunity, and their potential as therapeutic targets for HIV eradication strategies. 

## 2. Immune Checkpoints and Infectious Diseases

Since immune checkpoints play a crucial role in regulating immune responses against invading pathogens, their part in infectious diseases has been increasingly discussed in the last decade. T cells are persistently exposed to antigens in chronic infections resulting in their exhaustion. High PD-1 expression is correlated to an exhausted phenotype of T effector cells and, therefore, the PD-1 pathway has been particularly investigated in the context of chronic infections. So far, PD-1 has two known ligands, namely programmed death receptor ligand 1 (PD-L1) and PD-L2. Upon ligand binding, downstream signaling of PD-1 is induced resulting in various biological processes, such as inhibition of cytokine production, proliferation, and survival of T cells [2]. A variety of pathogens exploits PD-1 signaling to escape host antimicrobial immunity and those pathogens belong to different species including bacteria, protists, and animals [15,16]. In patients with active human tuberculosis (TB) caused by *Mycobacterium tuberculosis*, the number of PD-1 expressing cells in peripheral blood was elevated, and expression of PD-L1 was also up-regulated. During successful treatment of TB, expression of PD-1 and PD-L1 decreased in patients’ T cells and natural killer (NK) cells. Furthermore, ex vivo blockade of PD-1 or its ligands resulted in enhanced T cell effector function in isolated peripheral blood mononuclear cells (PBMCs) from actively TB-infected patients [17,18]. Thus, altered PD-1 signaling in TB infection might be one of the reasons for poor infection clearance, and anti-PD-1-therapy might be beneficial in addition to standard anti-TB-treatment [17]. Concerning protozoa infections, the unicellular parasite *Plasmodium falciparum* is the causing agent of malaria tropica. In Kenyan children who are persistently exposed to this pathogen, a high expression of PD-1 is observed in isolated effector memory CD8+ T cells, either alone or in combination with high expression of lymphocyte activation gene 3 protein (LAG3) [19]. LAG3 is another immune checkpoint molecule that is expressed on activated T cells as well as on NK cells and it down-modulates T cell immunity. By high-affinity binding to its receptor MHC II, LAG3 negatively regulates the expansion of T cells and controls the memory T cell pool size [20]. In addition to elevated PD-1 and LAG3 expression on CD8+ T cells, higher frequencies of CD4+ T cells expressing PD-1 are observed in malaria-infected children in Mali. In a murine malaria model, blocking PD-1 pathway in combination with blocking LAG3 signaling reduced parasitemia, improved T cell responses and improved antibody responses resulting in parasite clearance and extended survival of the mice [21]. No human trials with anti-PD-1 antibodies have been performed in malaria patients yet, but results from murine models seem promising. In worm infections, both *Schistosoma mansoni* and *Taenia crassiceps*, causing schistosomiasis or cysticercosis respectively, up-regulate PD-L1 expression on macrophages in mice during infection. In this way, T cell proliferation was inhibited in both diseases. Importantly, blockade of PD-L1 in schistosomiasis and blockade of PD-1, PD-L1, or PD-L2 in cysticercosis, blocked the ability of macrophages to induce T cell anergy and restored T cell proliferation in mice [22,23].

Several viruses including hepatitis B virus (HBV), hepatitis C virus (HCV), and HIV are responsible for chronic infectious diseases. Thus, it is not surprising that aberrant expression of immune checkpoints has been described in those aforementioned infections [16]. During chronic hepatitis B infection (CHB), PD-1 is considered as the dominant inhibitory receptor that can be targeted for therapy. However, up-regulation of CTLA4, T cell immunoglobulin and mucin 3 (TIM3), and other immune checkpoints on exhausted HBV-specific CD8+ T cells are also observed. TIM3 is a member of the immunoglobulin (Ig) superfamily and is expressed on cells of the innate and adaptive immune system, including CD4+ and CD8+ T cells. Although the exact mechanisms are not elucidated yet, the somewhat broad expression suggests a role of TIM3 in both innate and adaptive immunity with inhibitory effects on CD4+ and CD8+ T cell responses. Ex vivo or in vitro blockade of the PD-1, CTLA4, or TIM3 pathway restores impaired T cell responses. Thus, these molecules have the potential to act as indicators of T cell exhaustion and as therapeutic targets in CHB [24]. Similar results are observed in HCV infection, where CD4+ and CD8+ T cells in blood and liver from infected patients have high levels of PD-1 [25]. Furthermore, in a clinical evaluation of anti-PD-1 antibody Nivolumab (BMS-936558) in patients with chronic HCV infection, a modest response rate (HCV RNA reductions ≥0.5 log10 infectious units/mL in 5 out of 45 patients) and good tolerance are observed after single dose administration. However, the small number of patients included in this study makes it difficult to provide conclusive results. Although, spontaneous remission of chronic HCV is rare, one out of the nine patients within the placebo group also showed a clinical response. Therefore, a follow up study with an increased number of patients in each group and/or inclusion of multiple dose administration is required to clearly attribute the modest response rate of the Nivolumab-treated patient to PD-1 blockade. Even though the adverse events of Nivolumab treatment seemed mild to moderate in intensity and were mostly resolved in time without intervention, reduced immunological self-tolerance events (two patients with the onset of hyperthyroidism, one patient with a temporary conversion from hypothyroidism to hyperthyroidism, and one patient with exacerbation of adult onset diabetes) were reported in this study [26]. These self-tolerance events, which could be signs of autoimmune disease development, have to be carefully followed up as well.

Many studies concerning immune checkpoints in HIV have been conducted and are still ongoing today; of which selected findings will be discussed more in detail in the following sections. So far it can be concluded that the expression of PD-1 and other immune checkpoints might serve as prognostic biomarkers in tuberculosis, malaria, and chronic viral infections. In combination with standard drug treatment, blocking these pathways could be a major immunotherapeutic strategy in (functionally) curing the chronic infectious diseases, as is currently being developed for cancer therapies.

## 3. Programmed Cell Death Protein 1 (PD-1) Signaling in Human Immunodeficiency Virus (HIV) Immunity

**Figure 1:**
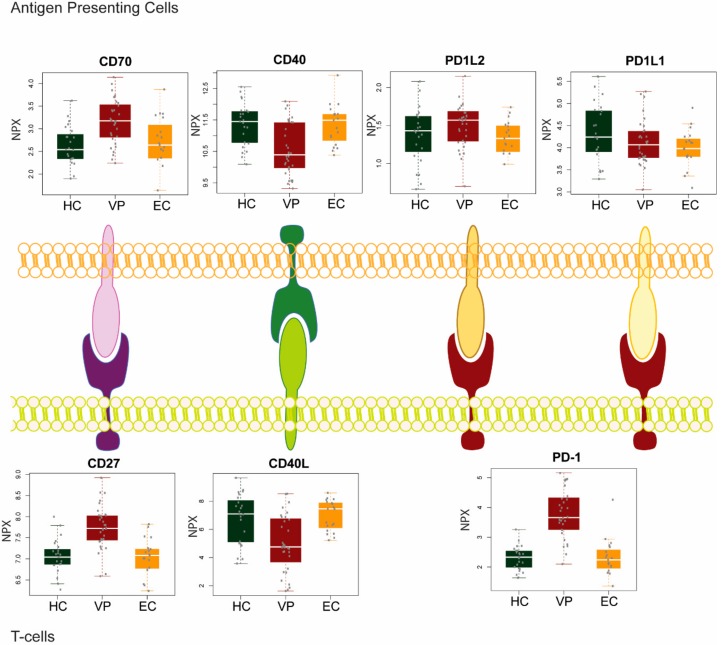
Plasma level of soluble ligand-receptor in two groups of human immunodeficiency virus 1 (HIV-1) infected individual viremic progressors (VP), and elite controllers (EC) and HIV-negative healthy control (HC).

In our recent study, we analyzed the plasma levels of secreted immune checkpoint molecules in two groups of HIV-1 infected patients with (viremic progressors; VP) or without viremia (elite controllers; EC) without any treatment and HIV-1 negative healthy controls (HC) [27] (Figure 1). In the inhibitory PD-1 pathway, there was a substantial increase of PD-1 level in VP compared to EC and HC (*p* < 0.001 in both the comparisons), but no difference was observed in PD-L1 between the groups, while there was a marginal decrease in PD-L2 in EC compared to VP (*p* = 0.016).

PD-1 has been determined as a major regulatory factor for T cell exhaustion in chronic HIV infection. Several studies reported that both the percentage of cells expressing PD-1 and the expression per cell on HIV-specific CD8+ T cells is elevated during chronic infection [28,29,30]. A higher level of the ligands PD-L1 and/or PD-L2 in plasma and/or immune cells in viremic HIV-infected individuals were observed. Sustained viremia and high Ag load might be responsible for the high levels of these molecules [31]. However, not only repeated Ag exposure plays a role in modulating the PD-1 pathway, but also the accessory HIV protein Nef can elevate PD-1 expression in vitro [32]. However, the PD-1 expression level correlates with disease progression, as measured by viral load and CD4+ T cell count, and might, therefore, be used as a disease severity marker [28,30].

Lower proliferation abilities were observed in HIV-specific CD8+ T cells expressing PD-1. Furthermore, these cells no longer expressed co-stimulatory receptor CD28 or perforin; and CCR7, as well as CD127, were only weakly expressed. These two latter molecules are essential for maintaining memory T cells. Besides, these CD8+ T cells that have very high PD-1 expression levels were more susceptible to death signals. Thus, high PD-1 expression might lead to a survival defect in vivo [15]. HIV-specific CD8+ T cells are not the only cell population where high PD-1 levels were observed during chronic HIV infection. Another vital T cell subset is CD4+ T helper cells, which coordinate both humoral and cellular immune responses. They are the primary target cells of HIV and these cells deplete over time when patients progress towards acquired immunodeficiency syndrome (AIDS) [33]. This results in inadequate pathogen-specific CD4+ T cell responses, including HIV-specific CD4+ T cell responses, and thus also in incomplete CD8+ T cell responses [34]. Additionally, PD-1 expression might have similar effects on both T cell subsets: up-regulation of PD-1 on HIV-specific CD4+ T cells has been observed and was positively correlated with viral load and inversely correlated with CD4+ T cell count. In lymph nodes of HIV-infected patients, PD-1 levels on CD4+ T cells were higher than in peripheral blood, and those cells were more similar to T follicular helper (Tfh) cells, which interact with B cells [35,36]. The latter form humoral immunity by producing antibodies. However, B cells are not solely affected by PD-1 signaling through Tfh cells, as they express PD-1 themselves upon activation [37]. The exact role of PD-1 in B cell activation, antibody production, and B cell dysfunction remains elusive, but a higher frequency of PD-1 expression has been observed on B cells in HIV-infected individuals [38]. Studies on simian immunodeficiency virus (SIV) suggest a role of PD-1 in depletion and survival of activated memory B cells. In vitro blockade of this pathway increases survival and proliferation of memory B cells in HIV infection and in vivo blockade in SIV-infected macaques increased the number of antibodies produced against SIV antigens. In concordance, stimulation with PD-L1 resulted in the deletion of activated B cells through apoptosis [39]. Interestingly, in HIV infection PD-L1 up-regulation has also been reported for B cells; thus, higher levels of PD-1 and its ligand could be a determining factor in B cell depletion. Additionally, expression of PD-L1 was also elevated on monocytes, macrophages, and dendritic cells (DCs) in HIV-infected subjects. PD-L1 expression might be induced by HIV-encoded Toll-like receptor (TLR) ligands and HIV accessory proteins, for example viral transcriptional transactivator (tat). Similar to PD-1, levels of PD-L1 positively correlate with viral load and inversely correlate with CD4+ T cell count [40,41,42]. Expression of PD-L1 is also induced on neutrophils during HIV infection and contributes to suppression of T cell functions [43]. Hence, a neutrophil-mediated immune suppression mechanism should be considered in HIV pathogenesis and disease progression.

It is assumed that chronic high viral antigen levels cause elevated PD-1 expression on HIV-specific CD8+ T cells and T cell exhaustion [28,30]. HIV-1-infected patients that are successfully treated with ART or highly active ART (HAART) show decreased PD-1 plasma levels and decreased PD-1 cell surface expression on HIV-specific CD8+ T cells. Thus, ART does not only reduce viral load but also allows for reconstitution of T cell effector functions. Furthermore, expression of PD-L1 on monocytes and DCs was lower in patients on ART compared with HIV-infected patients with progressive infection [40]. We and others made an interesting observation regarding PD-1 and its ligands in EC. This rare group of HIV-infected individuals can suppress viremia to undetectable levels while CD4+ T cell counts remain stable in the absence of ART. This indicates that EC have immunological mechanisms, possibly involving immune checkpoints, that efficiently suppress viral replication. Understanding these anti-HIV mechanisms within EC could be a key for a functional HIV cure [44,45]. These patients show similar expression patterns of PD-1, PD-L1, and PD-L2 as HIV-negative healthy controls [31]. This finding strengthens the idea that the PD-1 pathway plays a crucial role during chronic HIV infection. It is, therefore, not surprising that many studies have been performed where the effects of blocking the PD-1 pathway on HIV infection were assessed. In vitro or ex vivo blockade with anti-PD-L1 antibody results in increased proliferation capacity and cytokine production in HIV-specific CD8+ and CD4+ T cells [28,29,30,35,46]. Furthermore, PD-1 blockade results in improved B cell responses to HIV antigen. Also, blocking the interaction of PD-1 on follicular B helper T cells with PD-L1/2 on germinal center B cells during germinal center B cell differentiation increases immunoglobulin G (IgG) production [38,47]. Encouraging results were also obtained in studies on SIV infection using a non-human primate model. Not only B cell survival is improved when isolated PBMCs from SIV-infected macaques are cultured with PD-1 blocking antibody; enhanced antibody responses against both non-SIV-specific and SIV-specific antigens are observed when the PD-1 pathway is blocked in rhesus macaques in vivo, most likely because of enhanced B cell survival [39]. In addition to boosting humoral immunity, reflected by B cell proliferation and increased production of SIV envelope-specific antibodies, Velu et al. also observed enhanced cellular immunity when SIV-infected macaques were treated with anti-PD-1 antibodies. Virus-specific CD8+ T cells expanded rapidly and showed improved effector functions while the antibody treatment was well tolerated. Anti-PD-1 treatment was associated with reduced viral load and prolonged survival of the animals [48]. The therapeutic benefits of the PD-1 blockade observed in SIV-infected macaques raise the hope that the in vivo blockade of the PD-1 pathway could serve as a new approach to treat HIV-infected humans.

## 4. Altered Co-Stimulatory Pathways in HIV-1 Infection

We recently investigated the soluble protein levels of receptor-ligand pairs CD27/CD70 and CD40/CD40 ligand (CD40L) in VP, EC, and HIV-1 negative HC [27] (Figure 1). Our results showed distinct patterns among the different patient groups. While levels for both CD27 and CD70 were significantly increased in viremic progressors, CD40 and CD40L protein levels were significantly decreased compared to EC and HC (*p* < 0.01). Although both pairs constitute a co-stimulatory pathway, CD27/CD70 and CD40/CD40L signaling are affected in different ways while the virus is present in the body.

CD40L is a membrane protein expressed mainly by activated CD4+ T cells and interacts with its receptor CD40. Signaling through CD40L regulates both cellular and humoral immunity; it is required for the generation of CD8+ T cells, full T cell activation (both CD4+ and CD8+ T cells), chemokine production by macrophages and DCs, differentiation of B cells, and the IgM to IgG class switch. During HIV infection, CD40L appears to play multiple roles. It contributes to viral control during the acute phase of infection through (i) the induction of HIV-suppressive chemokines like macrophage inflammatory protein-1-alpha (MIP-1α), MIP-1β, and RANTES; (ii) the production of antibodies against HIV; and (iii) support of HIV-specific CD8+ T cells. At the same time, CD40L promotes HIV replication by increasing CD4+ T cell activation [49]. Contradictory results were obtained during later phases of infection. Several studies observed a decline in CD40L production and surface expression on CD4+ T cells during progressing disease and/or the development of AIDS. At the same time, CD4+ T cell effector functions, for example IL-12 production, were impaired [50,51,52]. Another study reports a lower percentage of CD40L-expressing lymphocytes in HIV-infected patients. However, from both HIV-infected and HIV-negative individuals, those CD4+ T cells that do express CD40L have similar CD40L protein levels on the cell surface [53]. Jenabian et al. found elevated plasma levels of soluble CD40L in ART-naïve chronic HIV-infected patients [54]. In contrast, in our studied cohort, plasma levels for both sCD40 and CD40L were low in VP compared to HC and EC [27]. Reduced CD40/CD40L signaling during HIV infection may be a factor for disease progression, and this might be mediated by actively suppressing CD40L surface expression by HIV. In this way, it could be a mechanism exploited by the virus to reduce expression of interferon gamma (IFNγ) and HIV-suppressive chemokines, as mentioned above, to produce a persistent infection. Furthermore, functional deficiencies of HIV-specific CD8+ T cells and their inability to control infection could partially be a consequence of CD40L downregulation since activated CD4+ T cells are essential for CD8+ T cell activity [49]. The study by Kaltenmeier et al. shows that these low CD40L-expressing T cells induce a shift in B cell differentiation from plasma cells to granzyme B-expressing B cells (GraB cells). These GraB cells, in turn, degrade the T cell receptor (TCR) zeta chain and subsequently suppress T cell proliferation [52]. Last, CD40L downregulation may render an HIV-infected patient more susceptible to opportunistic infections as similarities are observed between patients with AIDS and those having congenital CD40L deficiencies. Thus, therapies preventing and reversing the drop of CD40L/CD40 signaling might be useful and should be considered in HIV infection [49].

CD70 is transiently expressed on activated lymphocytes as a co-stimulatory molecule. Upon binding to its ligand CD27, CD70 can increase B and/or T cell activation. The interaction between CD4+ T cells and B cells via CD70/CD27 is crucial for B cell differentiation and Ig production. Usually, CD70 expression is transient to ensure maintenance of peripheral tolerance. However, during chronic immune activation CD70 is constitutively expressed on activated immune cells [55] and thus it is not surprising that we found elevated CD70 plasma levels in VP in our study [27]. As CD27 levels were also increased in VP compared to HC and EC, downstream signaling might be increased too. Such a chronic co-stimulation via CD70/CD27 was shown to lead to exhaustion of T cells, which may contribute to immune deficiency during HIV infection and AIDS [56]. In concordance with our study, Lantto et al. found higher frequencies of CD4+ CD70+ expressing T cells in HIV-infected individuals. The frequency correlated inversely with CD4+ T cell counts and correlated with B cell activation. Furthermore, CD4+ CD70+ T cell counts associated with expression of the first apoptosis signal receptor (Fas/CD95) on activated memory B cells and with plasma IgG levels. Hence, a link is suggested between abnormal activation of B cells and their impaired functionality resulting in hyperglobulinemia, increased sensitivity to apoptosis and, finally, accelerated memory B cell turnover during HIV infection [57]. Similar to abrogated CD40L signaling, increased CD70 signaling leads to impaired IgG responses as it strengthens IgM responses while preventing isotype class switch to IgG. Last, chronic CD70/CD27 co-stimulation in T cells impairs differentiation of germinal center B cells in a Fas-dependent manner [55].

Thus, it seems that reduced CD40 signaling and increased CD70 signaling could contribute to immune dysregulation in HIV-infected individuals with viremia.

## 5. Immune Checkpoints as Biomarkers in HIV-1 Infection

PD-1 constitutes the most studied immune checkpoint regarding HIV infection. However, interesting findings have been made for other immune checkpoint molecules. CTLA4, being the pioneer in immune checkpoint therapy in cancer, is much less appreciated in HIV and AIDS. Some groups reported an up-regulation of CTLA4 on HIV-specific CD4+ T cells that are associated with disease progression, i.e., correlating positively with viral load and negatively with CD4+ T cell count, and a reduced capacity of those cells to produce interleukin 2 [58,59]. Interestingly, CTLA4 was often co-expressed with PD-1 on HIV-specific CD4+ T cells isolated from untreated subjects, but heterogeneous expression patterns were observed on those cells isolated from elite controllers. Blocking the CTLA4 pathway alone or in combination with PD-1 blocking increased proliferation of HIV-specific CD4+ T cells in vitro, even though response rates differed greatly between studied subjects. Contrary to PD-1, CTLA4 expression decreased very slowly and only in a modest way after initiation of HAART; and increased again, as soon as HAART was interrupted [58]. Notably, CTLA4 was not up-regulated on HIV-specific CD8+ T cells [58,60].

Relatively little is known about the impact of LAG3 on HIV infection. A role in disease progression is proposed by Tian et al. who found elevated numbers of both activated CD4+ and CD8+ T cells expressing LAG3 and concomitant increased LAG3 density on these cells in HIV-infected subjects. Up-regulation of LAG3 on both T cell populations correlated positively with viral load and negatively with CD4+ T cell count. However, expression was not restricted to HIV-specific T cells as reported for PD-1 and CTLA4 [61]. Another group observed elevated LAG3 expression only on central memory CD8+ T cells in HIV-infected patients [60]. Still, LAG3 up-regulation could reflect persistent immune activation of T cells during infection, which is supported by the finding that LAG3 expression decreased again in HIV-infected individuals on successful ART [60,61]. Ex vivo blockade of LAG3 could enhance proliferation and effector functions of HIV-specific T cells. Interestingly, LAG3 and PD-1 were expressed on different T cell subsets in HIV-infected subjects, and only a small fraction of T cells (approximately 5%) co-expressed both immune checkpoints. Thus, LAG3- and PD-1-expressing T cells might represent two distinct populations of functionally impaired T cells in HIV infection [61].

As mentioned before, TIM3 has inhibitory effects on CD4+ and CD8+ T cell responses. Several studies indicate that TIM3 mainly restrains Th1-mediated immune responses by binding to Gal-9 [62], which is one of the multiple TIM3 ligands. In HIV-infected individuals, elevated TIM3 expression was observed on CD4+ and CD8+ T cells and this correlated with high T cell activation. Akin to LAG3, this increase is also associated with disease progression defined by viral load and CD4+ T cell count [63]. In our study, we noticed an up-regulation of TIM3 ligand Gal-9 in plasma of treatment-naïve HIV-infected individuals compared to HC and EC, suggesting that LAG3 signaling could be increased in viremic patients. Contradictory results have been described for TIM3 expression on HIV-specific T cells. High TIM3 levels were observed on HIV-specific CD4+ T cells [64], whereas negligible expression was found on HIV-specific CD8+ T cells [60]. However, Jones et al. reported increased TIM3 levels on HIV-specific CD8+ T cells in progressive HIV infection, and those cells showed a lack of proliferation markers and cytokine production in response to HIV-specific antigens ex vivo. Addition of an antibody blocking TIM3 signaling restored CD8+ T cell functionality and enhanced their expansion [63]. Heterogeneous observations in TIM3 signaling were also reported for HIV-infected individuals on ART. While we did not see a difference in Gal-9 protein levels in patients on long-term ART and healthy subjects (but higher levels in viremic progressors), Jones et al. reported a decline of TIM3 levels only in four out of seven patients on HAART. The remaining three subjects retained high protein levels of TIM3, which was associated with maintenance of high levels of T cell activation as well. Comparable to LAG3, the co-expression of TIM3 and PD-1 is somewhat atypical suggesting that distinct populations of T cells express either one or the other immune checkpoint molecule. In conclusion, TIM3 is described as a marker for an activated, but dysfunctional T cell population in HIV infection and its expression on (HIV-specific) lymphocytes is distinct from other immune checkpoints [63].

For each of the aforementioned immune checkpoints, up-regulation in protein levels is reported in HIV infection (Table 1). Whether this increase in expression of the inhibitory immune checkpoint molecules is a cause or a consequence of disease progression remains unclear. Also, studies on co-expression of these immune checkpoints as well as on how they influence or act with each other are scarce. One approach has been attempted where levels of PD-1, LAG3, and TIM3 were measured in patients with primary HIV infection followed by initiation of ART. After 48 weeks of successful treatment, therapy was interrupted (TI), and levels of the immune checkpoints and time until viral rebound were measured (as defined by viral load >400 copies/mL). The rationale was to find associations between biomarker levels and the time of viral rebound. Interestingly, none of the T cell exhaustion markers measured at TI predicted viral rebound. However, the pre-therapy levels of PD-1, LAG3, and TIM3 on CD4+ and the pre-therapy levels of PD-1 and LAG3 on CD8+ T cells were individual predictors for time to viral rebound. The levels of total HIV-1 DNA before treatment and at TI were also associated with shorter time to viral rebound, and correlations could further be established between HIV-1 DNA and biomarker levels before therapy. These results suggest that the size of the HIV reservoir is determined by T cell immune responses in early infection. When CD4+ and CD8+ T cells were evaluated for co-expression of PD-1, LAG3, and TIM3 before therapy, again only a minor percentage expressed a combination of two of those exhaustion markers, of which the highest percentage was observed for CD4+ T cells where 7.32% expressed both LAG3 and TIM3. An even lower percentage of CD4+ T cells, namely 2.3%, co-expressed all three immune checkpoints, supporting the hypothesis that T cells expressing different exhaustion markers are distinct populations [65].

The roles of the above presented immune checkpoints in HIV infection as well as effects of antibody-mediated blocking of these immune checkpoints are summarized in Table 1.

## 6. Immune Checkpoint Therapy in HIV-1 Infection

Even if more and more immune checkpoint inhibitors are approved by Food and Drug Administration (FDA) for cancer therapy, in vivo trials with patients suffering from viral infections, and in particular from HIV-1 are still scarce. As described above, in vitro and ex vivo studies where T cells or B cells were treated with blocking antibodies against either CTLA4, PD-1, LAG3, or TIM3 and subsequently challenged with HIV-1 or HIV-1 antigens showed promising results regarding restoring lymphocyte proliferation capacities and functions (Table 1). It is still unclear to which degree immune checkpoint blocking would reduce viral load and slow down disease progression in humans. A non-human primate model in rhesus macaques for in vivo blockade exists only for the PD-1 pathway, but not for other immune checkpoints. Astonishingly, next to reducing viral load, as described above, the administration of anti-PD-1 antibodies also showed beneficial effects in lowering microbial translocation and controlling opportunistic microbial infections in SIV-infected animals [66]. Thus, the application of PD-1 blocking antibodies increased overall immune responses resulting in prolonged survival of the macaques. In a subsequent study, the in vivo administration of repeated high-level doses of anti-PD-L1 antibody BMS-936559 in SIV-infected rhesus macaques provided a proof-of-concept for the safety. These rhesus macaques were also on ART at the beginning of the study, and when ART treatment was interrupted, two animals were able to maintain SIV RNA levels below detectable limits. Thus, blocking PD-L1 might lead to a restoration of immune function in SIV-infected rhesus macaques [67].

In human viral infections a clinical trial has been conducted that included patients with chronic HCV that were treated with PD-L1 antibody BMS-936558 (discussed above) [26]. Recently, a prospective first study of blocking PD-L1 in HIV-1-infected patients on successful combined ART was published [68]. This study included six men receiving a single low-dose infusion of BMS-936559 antibody and two men receiving placebo infusions. Although the primary clinical outcome was to assess safety, improvements in HIV-1-specific cellular immune responses were also evaluated. No grade three or higher adverse effects related to the antibody administration were observed in any of the participants. In two individuals, enhancement of HIV-1 specific responses was observed, illustrated by increasing percentages of gag-specific CD8+ T cells producing IFNγ. Additional support for a positive effect of BMS-936559 was provided by the observation of ex vivo proliferative responses of CD8+ T cells isolated from these two apparent responders to gag peptides. After day 28, CD8+ T cell responses declined again. During this time no change in either plasma or cell-associated HIV-1 RNA or cell-associated HIV-1 DNA was detected in any of the subjects. These rather modest responses may be due to the low dose administered that was chosen for safety reasons, but which might show little biological effect [68]. Nonetheless, a beneficial effect could still be observed in a subset of participants and, thus, blockade of the PD-1 pathway seems to have therapeutic potential for patients suffering from HIV-1 infection. Future studies should be performed with higher doses as well as with multiple dose administrations. Furthermore, approaches should be attempted in order to use several antibodies blocking different immune checkpoints to restore distinct exhausted cell populations (Table 1).

## 7. The Outlook of Immune Checkpoint Therapy

When it comes to immune exhaustion and the role of immune checkpoint molecules, the similarities between cancer and AIDS are quite astonishing. In both diseases, T cells are challenged with chronic antigen exposure that results in exhaustion of tumor- or HIV-specific T cells, respectively. Thus, immune cell functions are reduced and cells are consequently not able to target tumor or HIV-infected cells. This mechanism is likely to contribute to disease progression in both malignancies. The knowledge about immune checkpoints that was gained in the field of oncology could be used in the treatment of chronic viral infections, such as HIV. One of the advantages is that pharmacokinetic and toxicity profiles for drugs targeting immune checkpoints used in cancer therapy are already established. Therefore, the risks of severe side effects are reduced when these drugs are administered to HIV-infected patients [69]. A unique opportunity to study the impact of medications used in cancer therapy on HIV infection is also provided by a subgroup of HIV-infected patients that have developed cancer. For example, the effects of anti-CTLA4 antibody ipilimumab were studied in a single HIV-infected individual with concomitant metastatic melanoma [70]. After therapy, total memory T cells and cell-associated unspliced viral RNA copy numbers increased but no change in cell-associated HIV DNA was observed due to the sensitivity of the assay. This observation suggests that latently HIV-infected cells, which constitute an HIV reservoir, might have been reactivated and subsequently cleared by T cells [70]. In another study, two melanoma patients, a female with HCV mono-infection and a male with HIV-HCV co-infection, received pembrolizumab, but neither showed a decrease in HIV and/or HCV viral load [71]. In a third report of a 53-year-old man who received nivolumab, a PD-1 blocker used for advanced non-small cell lung cancer (NSCLC) reported no or little impact on HIV-1 replication or its reservoir [72]. However, there is a recent encouraging case report of a 51-year-old man, who smokes and has been HIV-infected since 1995, with stage IIIa NSCLC with epidermal growth factor receptor-/BRAF-/Kras-/programmed cell death ligand-1-characteristics. This patient received nivolumab and showed induction of synergistic ‘shock and kill’ mechanisms by transient reactivation of HIV replication within infected CD4+ T cells together with T cell activation and reduction of HIV reservoir [73].

Taken together, the idea of boosting endogenous immune responses as a potential therapy for cancer and chronic infections is being pursued by many researchers and promises benefits for affected patients. Thus, inducing HIV gene expression in latently infected cells followed by recognition and elimination of these cells by the immune system, e.g., via immune checkpoint therapy, could be explored as an additional way to achieve a functional cure in HIV-infected patients [69,74]. In vitro studies and the first in vivo studies on immune checkpoint therapy in HIV infection are very encouraging. Patients are likely to benefit the most from combination therapies, including drugs targeting different immune checkpoints, ART, and latency-reversing agents. However, one has to be careful when it comes to analyzing and targeting immune checkpoints on various immune cell subsets. It is especially vital to correctly quantify the proportions of phenotypic immune cell subsets expressing a particular receptor because bias might occur when analyses are performed on a whole cell subset, e.g., total CD4+ T cells instead of naïve and memory CD4+ T cells [8].

## Figures and Tables

**Table 1:** Information about the four major inhibitory immune checkpoint molecules in HIV infection.

Molecule	Expression	Function	Role in HIV Infection	Effects of Blocking Antibodies
Cytotoxic T-lymphocyte-associated protein 4 (CTLA4)	T cells	Regulates early stage of T cell activation	- Upregulation on HIV-specific CD4+ T cells and often co-expressed with PD-1	- Blocking CTLA4 pathway in vitro increased proliferation of HIV-specific CD4+ T cells
Programmed cell death protein 1 (PD-1)	T cells B cells Natural killer (NK) cells Monocytes Dendritic cells (DCs)	Limits the activity of immune cells in the periphery e.g., inhibits cytokine production, proliferation, survival of activated T cells	- Elevated numbers of cells expressing PD-1 and upregulated expression on HIV-specific CD8+ T cells elevated during chronic infection with loss of CD28 and perforin expression - Upregulation of PD-1 on HIV-specific CD4+ T cells - Upregulation of PD-1 and PD-L1 expression on B cells - Upregulated expression of PD-1 and ligand PD-L1 on monocytes, macrophages, dendritic cells, and induced on neutrophils	- In vivo blockade with anti-PD-L1 antibody increased proliferation capacity and cytokine production in HIV-specific CD8+ and CD4+ T cells - The first study of PD-L1 blocking in vivo: enhancement of HIV-1 specific responses by increasing percentage of gag-specific CD8+ T cells producing IFNγ in 2/6 individuals - In vitro blockade of PD-1 pathway increases responses, survival, and proliferation of memory B cells in HIV infection - In vivo blockade in SIV increased the amount of antibodies produced against SIV antigens
Lymphocyte-activation gene 3 (LAG3)	T cells NK cells	Negatively regulates T cell signaling and controls the memory T cell pool size	- Elevated numbers of LAG3-expressing CD4+ and CD8+ T cells with increased density of LAG3 on these cells	- Blocking LAG3 ex vivo enhances proliferation and effector function of HIV-specific T cells
T cell immunoglobulin and mucin-domain-containing-3 (TIM3)	Cells of innate and adaptive immune system	Regulating innate and adaptive immunity with both stimulating and inhibitory mechanisms	- Elevated TIM3 expression on CD4+ and CD8+ T cells - Upregulation on HIV-specific CD8+ T cells in progressive HIV infection with a lack of proliferation markers and cytokine production in response to HIV-specific antigens ex vivo - Negligible expression of TIM3 on HIV-specific CD8+ T cells	- Blocking TIM3 signaling ex vivo restored CD8+ T cell functionality and enhanced their expansion

## Abbreviations

PD-1	Programmed cell death protein
MHC	Major histocompatibility complex
APC	Antigen-presenting cell
TME	Tumor microenvironments
CTLA4	Cytotoxic T lymphocyte antigen 4
HIV	Human immunodeficiency virus
LRA	Latency-reversing agent
PD-L1/L2	Programmed death receptor ligand 1/ligand 2
TB	Human tuberculosis
NK cells	Natural killer cells
PBMCs	Peripheral blood mononuclear cells
LAG3	Lymphocyte activation gene 3 protein
HBV	Hepatitis B virus
HCV	Hepatitis C virus
CHB	Chronic hepatitis B infection
TIM3	T cell immunoglobulin and mucin 3
Ig	Immunoglobulin
VP	Viremic progressors
EC	Elite controller
HC	Healthy controls
Ag	Antigen
AIDS	Acquired immunodeficiency syndrome
Tfh	T follicular helper cells
SIV	Simian immunodeficiency virus
DC	Dendritic cell
TLR	Toll-like receptor
Tat	Transcriptional activator
ART	Antiretroviral therapy
HAART	Highly active antiretroviral therapy
MIP-1α	Macrophage inflammatory protein-1-alpha
MIP-1β	Macrophage inflammatory protein-1-beta
CD40L	CD40 ligand
IFNγ	Interferon-gamma
GraB cell	Granzyme B-expressing B cell
TCR	T cell receptor
Fas	First apoptosis signal receptor
TI	Therapy interruption
FDA	Food and Drug Administration
NSCLC	Non-small cell lung cancer
